# The Problem with Phi: A Critique of Integrated Information Theory

**DOI:** 10.1371/journal.pcbi.1004286

**Published:** 2015-09-17

**Authors:** Michael A. Cerullo

**Affiliations:** Cincinnati Institute for Cognitive Science, Cincinnati, Ohio, United States of America; Northwestern University, UNITED STATES

## Summary

In the last decade, Guilio Tononi has developed the Integrated Information Theory (IIT) of consciousness. IIT postulates that consciousness is equal to integrated information (Φ). The goal of this paper is to show that IIT fails in its stated goal of quantifying consciousness. The paper will challenge the theoretical and empirical arguments in support of IIT. The main theoretical argument for the relevance of integrated information to consciousness is the principle of information exclusion. Yet, no justification is given to support this principle. Tononi claims there is significant empirical support for IIT, but this is called into question by the creation of a trivial theory of consciousness with equal explanatory power. After examining the theoretical and empirical evidence for IIT, arguments from philosophy of mind and epistemology will be examined. Since IIT is not a form of computational functionalism, it is vulnerable to fading/dancing qualia arguments. Finally, the limitations of the phenomenological approach to studying consciousness are examined, and it will be shown that IIT is a theory of protoconsciousness rather than a theory of consciousness.

## Introduction

IIT is a novel new theory of consciousness proposed by Guilio Tononi [1–8]. IIT appears to be gaining popularity, and Tononi has recently teamed up with neuroscientist Chistof Koch to promote the theory [4,6]. IIT defines a property of a system called integrated information (Φ) and postulates that this is an exact measure of the quantity of consciousness of any system. Tononi defines integrated information as “the amount of information generated by a complex of elements, above and beyond the information generated by its parts” (Tononi [3], p. 216) and states, “The *integrated information theory (IIT)* of consciousness claims that, at the fundamental level, consciousness is integrated information” (Tononi [3], p. 217, italics in original). While the details of the mathematical calculation of integrated information have varied with each new version of IIT, this abstract definition of integrated information has remained unchanged [1–8]. The claim that consciousness is integrated information has also remained consistent through the various versions of IIT [1–8]. Tononi hopes to someday use measures of integrated information to determine the level of consciousness in patients who appear comatose [2–4]. Tononi and Koch have also claimed that, according to IIT, a neuromorphic electronic device with similar connectivity and dynamics to the brain would be conscious, while a human-equivalent emulation of a brain run on a Von Neumann digital computer would not be conscious [2–6,8].

While IIT should be lauded as an attempt to provide a precise empirical measure of consciousness, it ultimately fails in its stated goal. The main theoretical argument for IIT is the principle of information exclusion, which Tononi takes to be self-evident. It will be argued in this paper that information exclusion is unjustified and integrated information is not sufficient for consciousness. The empirical support of IIT will be challenged by the creation of an alternate theory of consciousness that has equal explanatory power to IIT but is based on a trivial property of conscious systems. Next, arguments will be examined from philosophy of mind showing that IIT is subject to dancing/fading qualia arguments. Finally, the fact that intuitively nonconscious systems can generate arbitrarily high values of Φ calls into question the epistemological approach of IIT and suggests that IIT is in fact a measure of protoconsciousness rather than consciousness.

## The Theoretical Foundation of IIT

The principal theoretical justification for IIT is the conjecture that, in perception, what is absent may be more significant to consciousness than what is present [2,3]. Tononi does not give a name for this conjecture, but it can be termed the principle of information exclusion. The concept of integration information, which is the foundation of all of IIT, is derived from the principle of information exclusion; hence, the validity of IIT rests on the correctness of information exclusion. In the discussion that follows, I will show that no valid arguments outside of Tononi’s intuition are advanced to support information exclusion. I will then challenge the self-evident nature of information exclusion as well as the relevance of integrated information to complex systems.

To begin his argument for information exclusion, Tononi explains that “integrated information theory (IIT) starts from phenomenology and makes use of thought experiments to claim that consciousness is integrated information” (Tononi [3], p. 216). He claims IIT is justified from “realizing that information and integration are the essential properties of our own experience” (Tononi [3], p. 217). Tononi asks us to imagine a single photodiode and a person both looking into a dark room [2,3]. We usually don't think of a single photodiode as having any consciousness, while we assume that if the person is awake, they are having significant conscious experience. To explain this difference, Tononi theorizes that when the photodiode “looks” at the dark room, it is only able to rule out one state, i.e., the room being light instead of dark. In the case of the awake person, Tononi states:

When you see the blank screen turn on, on the other hand, the situation is quite different. Though you may think you are performing the same discrimination between light and dark as the photodiode, you are in fact discriminating among a much larger number of alternatives, thereby generating many more bits of information. (Tononi [3], p. 218)

In other words, what makes the experience different is that when the person looks at a uniformly dark room, in addition to registering that the room is dark, they are able to rule out every other state they are capable of perceiving [2,3]. Tononi then describes this difference in the two systems in terms of information:

According to the IIT, the difference has to do with how much information is generated when that distinction is made. Information is classically defined as reduction of uncertainty: the more numerous the alternatives that are ruled out, the greater the reduction of uncertainty, and thus the greater the information. (Tononi [3], p. 217)

Unlike the photodiode, the person determines that there are not blue elephants running around the room nor anything else they could ever imaging seeing. All these things the person is not seeing amount to a very large amount of information. Returning to the photodiode example, Tononi states:

According to the IIT, it is all this added meaning, provided implicitly by *how* we discriminate pure light from all these alternatives, that increases the level of consciousness…the theory says that the more specifically one’s mechanisms discriminate between what pure light is and what it is not (the more they specify what light means), the more one is conscious of it. (Tononi [3], p. 218, italics in original)

Tononi is claiming that the level of consciousness is directly related to the amount of perceptual possibilities ruled out by the system. This is the principle of information exclusion. Tononi then uses this principle to define integration information:

This phenomenological analysis [of the photodiode thought experiment] suggests that, to generate consciousness, a physical system must be able to discriminate among a large repertoire of states (information) *and* it must be unified; that is, it should be doing so as a single system, one that is not decomposable into a collection of causally independent parts (integration). (Tononi [3], p. 219, italics in original)

Tononi does not provide any further arguments to justify information exclusion or his definition of integrated information but, instead, proceeds directly to formulate mathematical definitions of integrated information [1–8]. The problem is that I do not share Tononi’s intuition that consciousness is generated from ruling out alternatives. While the conscious systems we know best, mammalian brains, do discriminate from a large repertoire of states, this seems tautologically true because of the brain’s role in representing information rather than a major insight into the workings of consciousness. The intuition that integration is important for complex systems can also be challenged:

As humans, we seem to have the intuition that global integration of information is such a powerful property that no “simple” or “mundane” computational process could possibly achieve it. But our intuition is wrong. If it were right, then we wouldn’t have linear-size superconcentrators or LDPC codes. (Aaronson [9])

Therefore there is a large burden of proof on Tononi to provide arguments beyond his intuition to support information exclusion. Without further arguments, information exclusion cannot simply be taken as self-evident and there is no reason to suppose integrated information generates (or is) consciousness (i.e., integrated information is not sufficient for consciousness). Even if it is granted that the principle of information exclusion is correct, at most this would indicate that integrated information is necessary but not sufficient for consciousness [9,10].

## IIT 3.0

In the latest version of IIT, IIT 3.0, Tononi attempts to derive eight postulates of IIT from five axioms he claims come from phenomenology [8]. The principle of information exclusion is now adopted as axiom number three, the *axiom of information*, and is defined as:

Consciousness is information: each experience differs in its particular way from other possible experiences. Thus, an experience of pure darkness is what it is by differing in its particular way, from an immense number of other possible experiences. (Tononi [8], p.2)

While the first part of the axiom (that each experience is unique) seems consistent with phenomenology (when we take phenomenology to include both its use in analytical philosophy and the continental school of phenomenology), the second part of the definition (an experience is what it is by differing from an immense number of other possible experiences) is clearly Tononi’s addition of information exclusion to this axiom and is not a part of phenomenology [11–13].

The fifth axiom in IIT 3.0 is the *axiom of exclusion*, which states that:

Consciousness is exclusive: each experience excludes all others—at any given time there is only one experience having its full content, rather than a superposition of multiple partial experiences; each experience has definite borders. (Tononi [8], p.2)

Eric Schwitzgebel recently exposed a significant flaw when the exclusiveness of consciousness is expanded beyond its meaning in phenomenology [14]. Schwitzgebel points out that the axiom of exclusion seems to be Tononi’s (ad hoc) fix to the dilemmas created by IITs broad assignment of consciousness (e.g., IIT would assign consciousness to the entity of the United States) [14]. Yet the axiom of exclusion creates its own set of dilemmas; Schwitzgebel points out that according to IIT plus the axiom of exclusion, a person would immediately lose consciousness if a tiny organism was incorporated into their brain. This would occur because the system of the tiny organism plus brain would create a larger conscious entity which takes priority over the brain alone (remember, the experience having the “full content” takes precedence over partial experiences) [14]. Once again a property of phenomenology (that we have one experience at a time) is being extended beyond its meaning in phenomenology, with poor results. Ultimately the axiom of exclusion seems to be an untestable assertion: after all, how could we ever know if we were part of another conscious entity?

Axiom four of IIT 3.0, the *axiom of integration*, confounds integration with what phenomenologists call the unity of conscious experience [11–13]. The final two axioms, the *axiom of existence* and the *axiom of composition*, do seem consistent with the philosophical school of phenomenology, but not all philosophers would accept these two axioms [11,15]. Given the significant problems with the axioms of IIT 3.0, I will not discuss the eight proposed postulates further (however, see Aaronson [10] for a discussion of the problems with the postulates even if all the axioms are accepted).

## The Explanatory Power of IIT

Tononi claims there is significant empirical support for IIT and that it predicts the separation of consciousness in split-brain syndrome and the lack of consciousness in the cerebellum [1–8,16]. In split-brain syndrome, Tononi states:

…a “callosal” cut produces, out of a large complex corresponding to the connected corticothalamic system, two separate complexes, in line with many studies of split-brain patients (Gazzaniga, 2005). However, because there is great redundancy between the two hemispheres, their Φ value is not greatly reduced compared to when they form a single complex. (Tononi [3], p. 223)

Regarding the cerebellum, Tononi explains:

…its connectivity definitely suggests that the cerebellum is ill suited to information integration, since it lacks lateral connections among its basic modules. And indeed, though the cerebellum is heavily connected to the cerebral cortex, removing it hardly affects our consciousness, whereas removing the cortex eliminates it. (Tononi [16])

However, is IIT really providing any significant predictions in these cases? The explanatory power of IIT would be challenged if another theory based on an arbitrary potential property of consciousness analogous to integrated information could make these same predictions. Therefore I propose a theory of consciousness called Circular Coordinated Message Theory (CCMT). The justification for CCMT is the self-evident property that consciousness is related to information traveling in feedback loops within a system (the principle of information circulation). The amount of information circulation in a system, Omicron (O), is calculated by taking the information circulation of the system as a whole minus the information circulation in a minimal partition and is equal to the amount of consciousness in a system (O can also be said to *be* consciousness).The principle of information circulation is justified by thinking about why a single photodiode isn't conscious while a human brain is. Imagine both systems are viewing a dark room. The photodiode is not conscious because it doesn't convey any information in a circular pathway. On the other hand, when a human looks in the dark room, the sensory information is sent along many iterative cortico-thalamic pathways that circulate in the brain, and thus the human has a very high level of Omicron and consequently a high level of consciousness.

CCMT makes the same predictions as IIT for split-brain syndrome and the cerebellum. In split-brain syndrome CCMT predicts that two separate conscious systems emerge because each brain hemisphere contains significant cortico-thalamic loops. Because there is a large redundancy between the two hemispheres, their Omicron value is not greatly reduced compared to when they form a single complex. CCMT predicts that the cerebellum is not conscious because its modular design and lack of lateral connections among its basic modules make it ill-suited for circular information flow (wouldn’t a better answer for why the cerebellum is not conscious be that its function appears to be the support and coordination of planned movements rather than the cognitive functions central to consciousness such as executive function and attention?). Therefore, CCMT has as much empirical (and theoretical) support as IIT. The fact that a trivial theory of consciousness can be created that replicates the predictions of IIT challenges the explanatory power of IIT. It suggests the need for a more thorough analysis of what kind of predictions would provide empirical support without being trivially true for any theory as abstract as IIT.

Tononi also discusses two other types of research in support of IIT [3,8]. However, this research is merely consistent with IIT rather than evidence in support of the theory. The first type of research finds correlates of consciousness (in awake patients) using EEG and other measures and then attempts to use these measurements to develop consciousness detectors [17]. This type of research is empirically rather than theoretically driven. The second type of research examines broad measures of brain connectivity (e.g., effective connectivity) that are necessary for consciousness [18]. While it seems a reasonable hypothesis that communication across brain regions is necessary for consciousness, this type of research provides no specific support for IIT.

## IIT and Functionalism

Although it does not fit easily into the traditional classification of theories of consciousness, IIT appears to share traits with both identity and functionalist theories of mind [19]. Traditional identity theories claim that consciousness is identical to brain states, whereas IIT states that consciousness is correlated with or measured by the mathematically derived quantity Φ [1–8]. At times, Tononi emphasizes that integrated information is really two measures: Φ, which measures the intensity of consciousness, and the quale in qualia space that describes the specific subjective experience [1–8]. In either case, IIT appears to be identifying consciousness with a specific measure of information. In contrast, functionalism claims that it is the function and causal properties of the brain that are responsible for consciousness. Tononi attempts to add notions of causality in IIT 3.0 by using the Earth Mover’s Distance (EMD) equation to calculate Φ. Tononi claims the EMD equation allows the measurement of “internal causality” [8]. Even if we grant this is true, IIT is still not a theory of computational functionalism (as the name suggests, computational functionalism is a combination of functionalism and computationalism). Tononi implicitly states this when he claims “whether a system is conscious or not cannot be decided based on its input-output behavior only” (Tononi [8], p. 21). Tononi claims that a feed-forward program running the same computations as the human brain would not be conscious and acknowledges that IIT allows for functionally identical philosophical zombies [8,16].

Any theory such as IIT which rejects computational functionalism is vulnerable to one of the strongest arguments in philosophy of mind: fading/dancing qualia. Briefly, the fading qualia argument imagines that neurons are slowly replaced one by one with nanobots that perform the same function [20,21]. If computational functionalism is correct, then there will be no change in the person’s conscious experience as their brain is slowly replaced by machines because the system is always functionally identical. Now assume computational functionalism is incorrect; as the experiment continues, the subject’s consciousness will either slowly fade away or will suddenly cease after replacement of a single critical neuron. The sudden disappearance of all subjective experience with one neuron seems highly implausible. On the other hand, if consciousness slowly fades the subject will be aware of this, yet, because the new brain/machine is functionally identical to the original, they will be unable to act on or report this strange subjective experience. To show the same argument works with IIT, we keep the effector part of the nanobots and run the computations via radio transmission in a feed-forward algorithm run in a digital computer with a Von Neumann architecture. In this case, IIT would predict the neuron/nanobot hybrid has zero Φ: hence IIT would claim that the neuron/nanobot hybrid is not conscious and thus the subject would experience fading qualia.

The concept of dancing qualia is perhaps an even stronger argument against identity theories [20,21]. To create dancing qualia, the nanobots do not destroy the neurons but instead allow them to be switched on and off. As brain function switches between neuron and nanobot, computational functionalism would claim there is no change in subjective experience. In contrast, identity and non-computational functionalism theories would assert that particular qualia jump in and out of the subject’s experience as certain groups of neurons are turned on and off (i.e., they dance). Once again, because the system is always functionally equivalent, the subject is unable to act on these bizarre experiences. Similar to the fading qualia example, this argument works against IIT if we route the computations via radio transmission to a feed-forward algorithm run in a digital computer with a Von Neumann architecture. Fading and dancing qualia thought experiments show that identity and non-computational functionalist theories allow the unity of consciousness to be interrupted at any arbitrary point [22]. Given that one of the axioms of IIT 3.0 is the unity of conscious experience, IIT is especially vulnerable to fading/dancing qualia arguments [8].

## The Epistemology of IIT

Aaronson and Tononi recently engaged in an illuminating debate about the merits of IIT [9,10,16]. In the first round of argument, Aaronson challenged the coherency of IIT by showing that an expander graph generates arbitrarily large amounts of Φ [9]. In his response, Tononi granted that intuitively nonconscious systems could generate large values of Φ, and he and Aaronson agreed that something as uncomplicated as a network of XOR gates arranged in an *n x n* square grid could generate √*n* Φ [10,16]. By expanding the size of *n*, a network of XOR gates could be created with arbitrarily large values of Φ. Thus, a simple arrangement of XOR gates that does not perform any complex information processing can be made to have a higher Φ value than the human brain. Aaronson claimed that IIT’s seemingly absurd prediction in the XOR gate example shows the inconsistency of IIT [9,10]. Tononi responded to this criticism by challenging Aaronson’s intuition about consciousness [16]. Tononi claimed that it is intuitive to many people, including himself, that an XOR gate grid or a single photodiode can be conscious. Tononi’s response is not surprising given that IIT implies a form of panpsychism [1–8]. Tononi further argued that common sense cannot be used to challenge IIT since the theory is able to transcend intuition in its role as a fundamental theory of consciousness [16]. Tononi believes that we need a theory like IIT to determine consciousness in difficult cases such as photodiodes and XOR gates (or comatose patients) [3,16]. Finally, Tononi argued that we need to start from phenomenology to understand consciousness instead of beginning with the neural correlates of consciousness (NCCs) [3,8,16].

There are three central problems with Tononi’s response to Aaronson. The first problem is related to Tononi’s phenomenology-first approach to studying consciousness. This is not to deny the role of phenomenology in the scientific understanding of consciousness. However, people can come to very different conclusions about the fundamental properties of consciousness based on their own subjective experiences [11]. Thus Aaronson and Tononi can disagree about the very possibility of a photodiode having subjective experience. It is not clear how this disagreement can be resolved. Yet Aaronson is certainly correct in pointing out Tononi’s error in claiming that the consciousness of a photodiode and an XOR gate grid (and the lack of consciousness in the cerebellum) are both evidence in favor of and predictions of IIT [10]. Rather than starting with difficult cases like photodiodes or the cerebellum, Aaronson suggested we must develop a theory of consciousness using paradigm cases, which are generally believed to have some level of conscious (e.g., the cerebral cortex) [10].

The second problem with Tononi’s response is that he is not using the word consciousness in the same way as Aaronson. Aaronson suggested they must be using the word differently since he could not even conceive of what would count as a counterexample to IIT if the absurdity of an XOR gate grid with human-level consciousness is rejected by Tononi [9,10]. Given that Tononi does reject this counterexample, how can we reconcile their disagreement? It turns out that Aaronson is interested in the type of subjective experience humans and animals have when awake, which guides their behavior and which they lack when asleep or anesthetized [10]. Aaronson’s use of consciousness is therefore consistent with the way the term is used in cognitive neuroscience, psychology, and neurology/psychiatry (and also with the way most philosophers talk about consciousness) [23–37]. In contrast, IIT is a theory of a much more general form of subjective experience and, hence, measures protoconsciousness rather than consciousness. In his discussion of IIT and panpsychism, Tononi explains:

How close is this position to panpsychism, which holds that everything in the universe has some kind of consciousness? Certainly, the IIT implies that many entities, as long as they include some functional mechanisms that can make choices between alternatives, have some degree of consciousness. Unlike traditional panpsychism, however, the IIT does not attribute consciousness indiscriminately to all things. (Tononi [3] p. 236)

IIT is actually a version of panpsychism known as panexperientialism [38–40]. The difference between the two theories is that “rather than claiming that everything has a mind, or even that everything has some species of mentality, the panexperientialist claims only that everything has experience” (Kind [41] p. 3). Rosenberg describes panexperientialism as “the view that experience exists throughout nature and that mentality (i.e., a thing requiring cognition, functionally construed) is not essential to it” (Rosenberg [39] p. 91). Protoconsciousness (or proto-mentality) is thus defined as experience separated from cognition or mind (not to be confused with Chalmers’ use of protoconsciousness to refer to the consciousness of the most basic entities in the universe, such as quarks [42]). Theories of panexperientialism therefore measure protoconsciousness (or proto-mentality) rather than consciousness. Given that IIT claims only some systems (i.e., those with non-zero Φ) generate protoconsciousness, it is more accurately described as a theory of partial-panexperientialism. Tononi does not distinguish between protoconsciousness and consciousness and argues that the subjective experience of a single photodiode and human are fundamentally the same, differing only in their intensity and their quale:

IIT claims that consciousness is not an all-or-none property, but is graded: specifically, it increases in proportion to a system's repertoire of discriminable states. Strictly speaking, then, the IIT implies that even a binary photodiode is not completely unconscious, but rather enjoys exactly 1 bit of consciousness. Moreover, the photodiode's consciousness has a certain quality to it—the simplest possible quality—that is captured by a single q-arrow of length 1 bit. (Tononi [3], p. 236)

It is far from clear what type of subjectivity could be shared by a single photodiode and a fully conscious human being, and this is a major part of the ongoing debate about panpsychism [38–46]. Rosenberg suggests protoconscious experiences have no mind associated with them and that “the experiences we might attribute to noncognitive systems do not contain ‘little pains’ or ‘little specks of blue’ but instead have some kind of qualitative character very alien to us” (Rosenberg [39] p. 96). Thankfully, we do not need to resolve the debate about panpsychism and panexperientialism to understand some differences between the subjectivity of a human and a photodiode. A photodiode and an XOR gate grid lack the cognitive properties associated with the function of consciousness in mammalian brains. In mammalian brains, consciousness is *associated* with awareness, memory, and executive function [23–37]. Consciousness, or at least the cognitive features that consciousness is correlated with, appears to have evolved in mammals to allow for the global access of information in the planning and initiation of behavior [25,37,47,48]. It is precisely this type of consciousness within mammalian brains that Aaronson is interested in understanding. Of course, animals may have all sorts of protoconscious experiences going on in their cerebellum or even within parts of the cerebral cortex, but we have no way to verify the existence of these subjective experiences outside of cognition.

The type of consciousness that Aaronson suggests scientists who study consciousness are most interested in [9,10] can be labeled cognitive-consciousness, while the type of consciousness measured by IIT can be labeled noncognitive-consciousness. It may be noticed that the division of consciousness into cognitive and noncognitive aspects bears a resemblance to Block’s division of consciousness into access (A) and phenomenal (P) consciousness [49]. However, in Block’s division, A and P consciousness are both part of cognitive-consciousness in the sense that both of these types of consciousness are associated with an entity with a cognitive structure (the self or mind). Block defines A and P consciousness as:

The paradigm P-conscious states are sensations, whereas the paradigm A-conscious states are "propositional attitude" states such as thoughts, beliefs, and desires, states with representational content… (Block [49], p. 232)

At this point it is worth pausing for a moment to examine our rapidly growing catalogue of conscious experiences. Combining all of the variations gives us a taxonomy of four types of consciousness: Chalmers’ primitive protoconsciousness (e.g., the consciousness of quarks), Rosenberg’s protoconsciousness or noncognitive-consciousness (e.g., the consciousness of a photodiode or XOR gate grid, also termed microexperience by Strawson [50]), Block’s access-cognitive-consciousness (i.e., consciousness associated with representational content), and Block’s phenomenal-cognitive-consciousness (i.e., consciousness associated with sensations). Using this terminology, we can see Tononi is claiming that a photodiode and an XOR grid are *noncognitively* conscious, while Aaronson is claiming they are not (access or phenomenally) *cognitively* conscious. Thus their respective claims about the photodiode and XOR gate grid are not incompatible. Given that when most neuroscientists and psychologists (to say nothing of neurologists!) speak of consciousness they have cognitive-consciousness in mind, it seems fair to say the default use of the term consciousness refers to cognitive-consciousness [23–37]. Typical examples of theories of cognitive-consciousness are Baars’ global workspace model of consciousness [51–53] and Dehaene and Naccache’s [30] workspace framework of consciousness. These theories attempt to understand consciousness in the context of its cognitive functions. The type of consciousness measured by IIT should be differentiated from these theories by labeling it with the prefix “proto-” or calling it “noncognitive.”

Any theory of proto- or noncognitive-consciousness such as IIT faces concerns about being either implausible or irrelevant [40]. Kind suggests that the concept of noncognitive-consciousness is incoherent because it requires separating experience from cognition and thus requires the existence of experiences independent of experiencers [41]. Even if noncognitive-consciousness is a coherent concept, it is still likely to be irrelevant to the study of consciousness [40,41]. The relationship (if any) between noncognitive-consciousness and cognitive-consciousness appears to be a mystery and is not specified within IIT. Rather than clarifying the nature of consciousness, the addition of protoconsciousness seems to instead add another layer of mystery to the enigma of consciousness [40]. Nagasawa further suggests, “If properties of protoconsciousness are essentially different from that of properties of consciousness, protoconsciousness has nothing to do with ordinary consciousness” (Nagasawa [40] p. 6). In the case of IIT this can be most clearly illustrated by going back to Tononi’s original motivation: understanding consciousness in difficult cases. What if IIT showed that a part of the nervous system not associated with cognitive-consciousness, say the enteric nervous system, had a high level of Φ (noncognitive-consciousness)? Should doctors then refuse to remove the vagus nerve (a central part of the enteric nervous system) to save the patient’s life? What would we make of the measurement of a high level of Φ in someone in a neuro-vegetative state? Are they conscious in the sense we care about: i.e., are they suffering pain, in a dreamlike state, or fully awake? Or are they simply highly noncognitively conscious? In the latter case, we are no better off in understanding what is going on inside their head than before we measured Φ. Without an ability to differentiate cognitive from noncognitive-consciousness, IIT seems to fail in its original goal of understanding consciousness in difficult cases, as these are precisely the cases in which determining *cognitive-*consciousness is difficult.

The final issue with Tononi’s response to Aaronson is his misunderstanding of the hard problem of consciousness. Tononi states that:

While identifying the “neural correlates of consciousness” is undoubtedly important, it is hard to see how it could ever lead to a satisfactory explanation of what consciousness is and how it comes about. (Tononi [8], p 2)

Again Tononi states:

As David Chalmers has argued convincingly, the problem of going from the brain to experience is indeed so hard that it may actually be impossible to solve. On the other hand, argues IIT, it may be hard, but not impossible to go from experience to the brain (or to expander graphs). (Tononi [16], underlining in original)

To truly explain consciousness would require solving what Chalmers calls the hard problem of consciousness [20,54]. According to Chalmers, the easy problem of consciousness is explaining *how* the brain generates the behavior associated with consciousness. In contrast, the hard problem requires a theory to address the question of *why* any physical process generates (or is) consciousness [20,54]. Even if IIT is correct, it does not explain *why* integrated information generates (or is) consciousness (to be fair, neither computational functionalism nor the identity theory can solve the hard problem of consciousness). IIT also does not appear to offer any insight into the easy problem of consciousness. Instead, what IIT attempts to address is what Chalmers and Aaronson call the Pretty Hard Problem (PHP) of consciousness [9,10]. The PHP of consciousness is the problem of predicting which physical systems give rise to consciousness. Given the prior discussion, IIT could at best only address the PHP of noncognitive-consciousness. It also seems implausible that the easy problem and PHP of consciousness are unrelated, as IIT implicitly asserts.

## Conclusion

The goal of this paper was to demonstrate how IIT fails at its stated goal of quantifying consciousness. The main theoretical argument for IIT is the principle of information exclusion. Yet there is no evidence in support of information exclusion beyond Tononi’s claim that it is self-evident, and consequently integrated information does not appear to be sufficient for consciousness. IIT also fails to exhibit any explanatory power given that a trivial theory of consciousness, CCMT, was able to make the same predictions. IIT is not a computational functionalist theory of consciousness and is therefore vulnerable to fading/dancing qualia arguments. The fact that intuitively nonconscious systems can generate arbitrarily high values of Φ suggests that IIT is a theory of proto- or noncognitive-consciousness that says nothing about the type of consciousness discussed by most neuroscientists and psychologists. Finally, IIT seems to be a theory addressing the pretty hard problem of consciousness rather than the hard problem of consciousness.

The declaration that consciousness is Φ is an extraordinary claim, and as Carl Sagan suggested, extraordinary claims require extraordinary evidence. Therefore, there is a large burden of proof on proponents of IIT to justify the relevance of information exclusion and the relationship between integrated information and consciousness. While IIT should be lauded as an attempt to provide a precise empirical measure of consciousness, it ultimately fails in its stated goal. Researchers in artificial general intelligence and artificial consciousness can be reassured that IIT does not banish the ghosts from their machines. After Tononi presented a talk on IIT at the 2014 Science of Consciousness meeting, Ned Block raised his hand and stated, “You have a theory of something, I am just not sure what it is [55].” I would suggest that IIT is a theory of partial-panexperientialism that, even if correct, does not help us to understand or predict the kind of consciousness that is relevant to our subjective experience.
